# Acute Myeloid Leukaemia Driven by Rare HNRNPH1::ERG Fusion Gene in an Adult, With Distinct Transcripts Detected by RNASeq at Diagnosis and Relapse: A Case Report

**DOI:** 10.1002/jha2.70359

**Published:** 2026-09-15

**Authors:** Hannah Al‐Yousuf, Pablo Prieto, Robert Baker, Katherine Clesham, Kate Xu, Andrew Wilson, Jiexin Zheng, Rajeev Gupta, Rob Sellar, Jenny O'Nions

**Affiliations:** ^1^ Department of Haematology University College London Hospital NHS Foundation Trust London UK; ^2^ Specialist Integrated Haematology Malignant Diagnostic Service Manual Blood Sciences HSL Pathology London UK; ^3^ UCL Cancer Institute London UK

**Keywords:** fusion gene, fusion transcript, leukaemia, myeloid leukaemia, myeloid

## Abstract

Acute myeloid leukaemia (AML) cases with rare fusion genes, such as ERG‐related fusions, constitute less than 1% of AML. They often present with a normal karyotype by conventional cytogenetics but are increasingly detected by RNA sequencing (RNAseq), although their characteristics and the utility of fusion genes as an MRD marker are poorly defined. We report a case of a 19‐year‐old female presenting with *HNRNPH1::ERG* AML, the fifth reported case to our knowledge, who developed a second *HNRNPH1::ERG* fusion at relapse that was not detectable on the original MRD assay. This case highlights potential mechanisms driving disease recurrence and underscores the importance of characterizing MRD kinetics and transcript dynamics to inform prognosis and therapeutic decisions. We highlight the benefit of incorporating RNAseq into testing both at diagnosis and at relapse, and the need to further characterise MRD kinetics for rare fusion genes in AML.

**Trial Registration:**

The authors have confirmed clinical trial registration is not needed for this submission

1

Fusion genes are present in 30%–40% of acute myeloid leukaemia (AML) [1] and play a key role in leukemogenesis. Their identification is pivotal in AML classification and can provide disease‐specific biomarkers to monitor measurable residual disease (MRD) [2]. Fusion genes involving *ERG* (ETS‐related gene, on chr 21q22) make up < 1% of AML and are classified as “AML with other rare recurring translocations” [3]. They typically have an apparently normal karyotype using conventional cytogenetics but are detectable by RNA sequencing (RNAseq) and are increasingly identified as genomic platforms improve [4]. However, their prognostic significance is unclear and their use in MRD monitoring is not standardised. We describe a case of AML driven by a novel *HNRNPH1::ERG* [t(5;21)(q35;22)] fusion in a young adult, who developed a second *HNRNPH1::ERG* fusion gene at relapse. This case highlights potential mechanisms of relapse and the need to further characterise MRD kinetics for rare fusion genes in AML.

A 19‐year‐old female presented to the Emergency Department with fever and pancytopenia (Hb 60 g/L, WCC 1.8 × 10^9^/L, neutrophils 0.1 × 10^9^/L, platelets 7 × 10^9^/L). Bone marrow aspiration confirmed AML with 89% blasts (CD34+CD117+ HLADR−CD33+ CD13+CD7+CD56wk, CD15wkCD38+MPO+ by flow cytometry, Beckman Coulter). No abnormalities were detected by fluorescence in‐situ hybridisation ([FISH], Cytocell) or molecular karyotyping by chromosomal microarray (8 × 60K oligonucleotide arrays, Agilent); however, RNAseq (Table S1a) demonstrated an *HNRNPH1::ERG* fusion in 88% of reads between *HNRNPH1* exon 14 and *ERG* exon 11 (breakpoint chr 5:179614341, chr 21:38383923), with no other mutations detected (Table S1b). A qRT‐PCR assay was designed with primers to the fusion sequence for MRD monitoring.

The patient received induction treatment with DA (3 + 10) (Daunorubicin 60 mg/m^2^, Days 1, 3 and 5 + cytarabine 100 mg/m^2^ BD for 10 days) and one dose of gemtuzumab‐ozogamicin 3 mg/m^2^. Post‐induction bone marrow showed complete remission (CR), with no detectable *HNRNPH1::ERG* transcripts. She received a second induction cycle of DA (3 + 8) and two cycles of consolidation with high‐dose cytarabine (3 g/m^2^ BD Day 1, 3 and 5). Bone marrow biopsies after each cycle showed flow cytometric and molecular MRD‐negative CR.

Post end‐of‐treatment (EOT), the patient underwent clinical monitoring due to lack of a suitable donor, with three‐monthly bone marrow biopsies. Morphological MRD‐negative CR was confirmed at 3 months post‐EOT; however, she relapsed at 6 months with 28% blasts by flow cytometry. Cytogenetic analysis detected a newly acquired 1q gain. Molecular testing using the MRD assay developed at diagnosis was negative. RNAseq identified a second *HNRNPH1::ERG* fusion, between *HNRNPH1* exon 4 and *ERG* exon11 (breakpoint chr5:179621034, chr21:38383923) (Figure S1). A qPCR assay for the new *HNRNPH1::ERG* fusion was designed. Prior samples with sufficient remaining material (EOT and 3 months post‐EOT) were retrospectively tested, demonstrating detectable transcripts of the exons 4:11 fusion gene, consistent with expansion of this clone after treatment (4.27e‐5 *HNRNPH1::ERG* transcripts at EOT, rising to 4.33e‐3 transcripts at 3 months post‐EOT, Figure 1).

**FIGURE 1:**
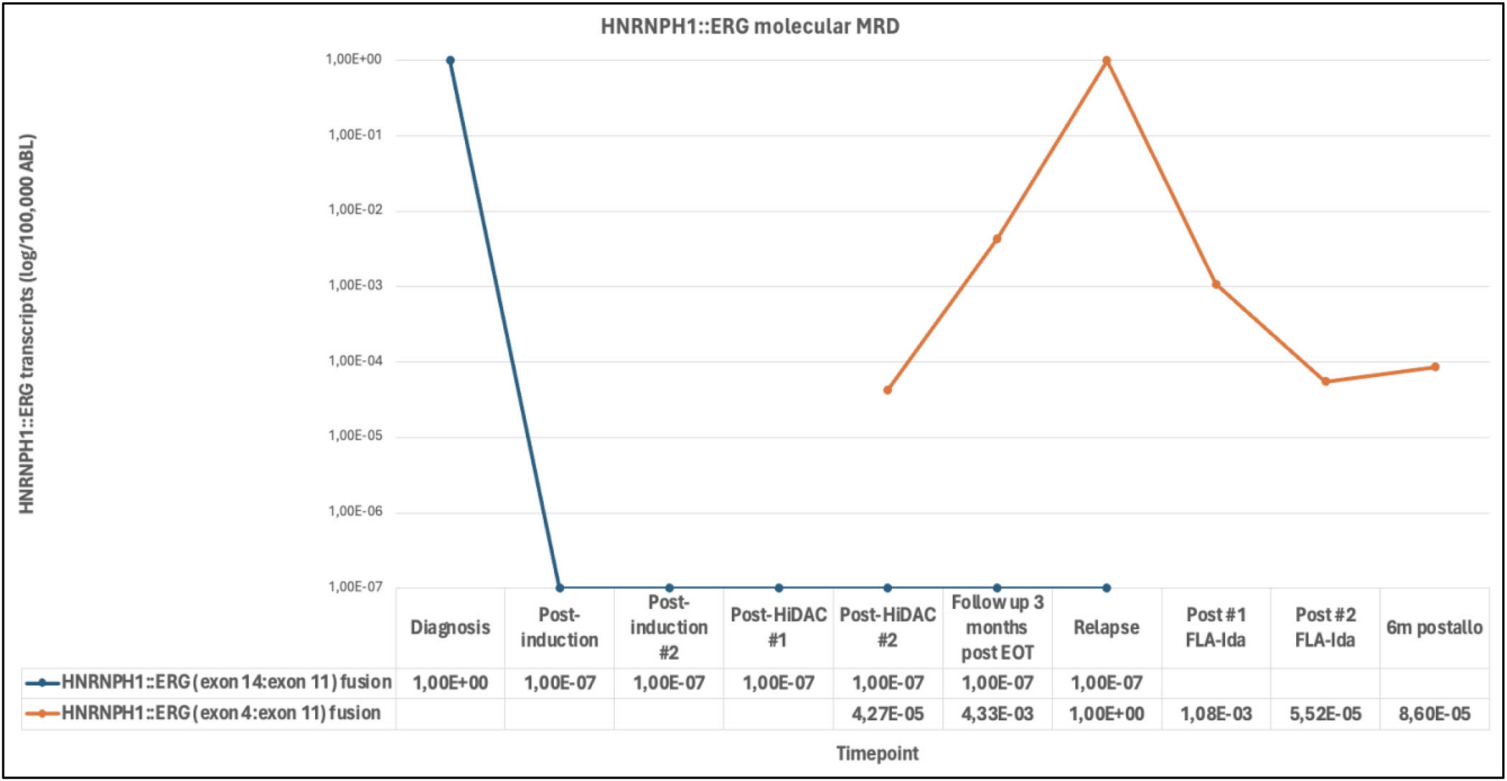
Molecular MRD measurement of *HNRNPH1::ERG* fusion transcripts by quantitative RT‐PCR over the patients’ disease course. *HNRNPH1::ERG* fusion (exons 14:11) transcripts detected at diagnosis and *HNRNPH1::ERG* (exons 4:11) detected at relapse and retrospectively tested are shown as log‐fold changes from baseline.

She received two cycles of FLA‐IDA (Fludarabine 30 mg/m^2^, Days 1–5, Cytarabine 2000 mg/m^2^, Days 1–5, Idarubicin 8 mg/m^2^, Days 1–3), achieving morphological CR and reduction in molecular‐MRD from 1.08e‐3 to 5.52e‐5 *HNRNPH1::ERG* transcripts. She proceeded to a 10/10 matched unrelated donor myeloablative (Flu/CyTBI) allogeneic stem cell transplant (alloHSCT), recovered successfully and remains under monitoring at 12 months post‐alloHSCT.

Heterogeneous nuclear ribonucleoprotein H1 (*HNRNPH1*), on chromosome 5q35 [5], plays a key role in RNA splicing and transcriptional regulation. *ERG* is involved in haematopoietic regulation and is overexpressed in numerous malignancies, including AML [6], where it is seen predominantly in paediatric patients [7]. The best characterised *ERG*‐rearranged AML, t(16;21)(p11;q22) (*FUS::ERG*), conveys shorter median overall and disease‐free survival, and higher relapse rates after alloHSCT [8, 9]. Evidence for the prognostic use of *FUS::ERG* as an MRD marker is limited to case series, but correlation between transcript levels and outcomes are reported [9, 10].

Only five cases of *HNRNPH1::ERG* AML are described to date [5, 11, 12], with median age of 9 years (range 4–54), including two adults aged 42 and 54. The *HNRNPH1*::*ERG* protein shares the same ETS binding domain as *FUS*::*ERG*, so it is hypothesised that leukemogenesis of *HNRNPH1*::*ERG* AML is similar; namely, transcriptional repression resulting in deregulation of cell differentiation, proliferation and apoptosis [5]. Characteristic features of *HNRNPH1::ERG* AML and similarities between *HNRNPH1::ERG‐* and *FUS::ERG*‐driven AML are undefined. In concordance with existing literature, our patient had no abnormalities detected by conventional cytogenetics, highlighting the need to employ RNAseq to identify rarer fusions. CD56 expression was detected on leukaemic blasts in our case, contrasting with immunophenotypes reported in previous *HNRNPH1::ERG* cases but previously reported in *FUS*::ERG [13]. All previously described *HNRNPH1::ERG* cases were refractory to induction chemotherapy, with poor outcomes (median OS of 8 months [3–60]) and only two patients achieving alloHSCT after salvage chemotherapy. Our patient suffered early relapse after chemotherapy, suggesting alloHSCT may be required for longer‐term survival in these patients.

Three different *HNRNPH1::ERG* transcripts have been described in AML, resulting in increased *ERG* expression in leukaemic cells. Lu et al. describe an *HNRPH1* exon 4:*ERG* exon10 fusion [11]. Jiang et al. identified two transcripts present in the same patient at diagnosis [5]; *HNRPH1* exon 3:*ERG* exon 11 and *HNRNPH1* exon 2:*ERG* exon 11. Our case also had two distinct *HNRNPH1::ERG* fusion genes, one detected at diagnosis (*HNRNPH1* exon14:*ERG* exon11) and the second at relapse (*HNRPH1* exon 4: *ERG* exon 11). The detection of two different variants of *HNRNPH1::ERG* during treatment could be accounted for by: (1) a subclonal population present at AML diagnosis, below the limit of detection of current sequencing platforms, which became the dominant clone due to selection pressures; (2) clonal evolution with acquisition of a new fusion gene during treatment.

We report the third adult case of *HNRNPH1::ERG* AML, and the first to describe a different fusion transcript detected at relapse and report the dynamics of *HNRNPH1::ERG* transcripts. Our case highlights that its use as an MRD marker is not fully elucidated and advises caution when using rarer fusion genes as markers of MRD, as their behaviour is not well‐characterised. In addition, those involving genes encoding several splice variants or isoforms may result in different fusion transcripts within the same patients’ AML. This case highlights the benefit of incorporating RNAseq into testing both at diagnosis and at relapse. Improvements in RNASeq assay sensitivity may enable earlier detection of relapse driven by rare fusion genes and further understanding of the mechanisms of disease resistance to treatment.

## Author Contributions

**Hannah Al‐Yousuf**: data curation, formal analysis, investigation, writing – original draft, writing – review and editing. **Pablo Prieto**: data curation, formal analysis, investigation, writing – original draft. **Robert Baker**: methodology, writing – review and editing. **Katherine Clesham**: writing – review and editing. **Kate Xu**: writing – review and editing. **Andrew Wilson**: writing – review and editing. **Jiexin Zheng**: writing – review and editing. **Rajeev Gupta**: writing – review and editing. **Rob Sellar**: supervision, writing – review and editing. **Jenny O'Nions**: conceptualisation, supervision, investigation, formal analysis, writing – original draft, writing – review and editing.

## Funding

The authors have nothing to report.

## Consent

Written informed consent was obtained from the patient for the publication of this case report (attached).

## Conflicts of Interest

The authors declare no conflicts of interest.

## Supporting information




**Supporting information file**: jha270359‐sup‐0001‐SuppMat.docx

## Data Availability

Data that support the findings of this study are available upon reasonable request from the corresponding author.
