# Supplementary material for: Acute Myeloid Leukaemia Driven by Rare HNRNPH1::ERG Fusion Gene in an Adult, With Distinct Transcripts Detected by RNASeq at Diagnosis and Relapse: A Case Report
Source: EJHaem. 2026 Sep 15;7(5):e70359. doi: 10.1002/jha2.70359 (PMC13575624; doi:10.1002/jha2.70359)
Supplement: Supplementary file 1 [file JHA2-7-e70359-s001.docx]

**Supplementary Figure 1*.*** *HNRNPH1::ERG* fusion sequences detected in the patient at diagnosis and relapse. Two different fusion sequences from *HNRNPH1* gene (with breakpoints in exons 14 and 4) were fused to the same exon 11 sequence of *ERG*.

***HNRNPH1* exon 14 fusion sequence**

ATGGGAAAAATTTGAGACGCAATACCAATACTTAGGATTTTGGTCTTGGTGTTTGTATGAAATTCTGAGGCCTTGATTTAAATCTTTCATTGTATTGTGATTTCCTTTTAGGTATATTGCGCTAAGTGAAACTTGTCAAATAAATCCTCCTTTTAAAAACTGCA

Genomic loci: **chr 5:179614341 (canonical** transcript ID ENST00000393432.9**).**

***HNRNPH1* exon 4 fusion sequence**

ATTCAAGTCAAACAACGTTGAAATGGATTGGGTGTTGAAGCATACTGGTCCAAATAGTCCTGACACGGCCAATGATGGCTTTGTACGGCTTAGAGGACTTCCCTTTGGATGTAGCAAGGAAGAAATTGTTCAGTTCTTCTCAG

Genomic loci: **chr 5: 179621034 (canonical** transcript ID ENST00000393432.9**).**

**ERG exon 11 fusion sequence**

GCAGTGGCCAGATCCAGCTTTGGCAGTTCCTCCTGGAGCTCCTGTCGGACAGCTCCAACTCCAGCT GCATCACCTGGGAAGGCACCAACGGGGAGTTCAAGATGACGGATCCCGACGAGGTGGCCCGGCGCTGGGGAGAGCGGAAGAGCAAACCCAACATGAACTA

Genomic loci **chr 21:38383923 (**transcript ID ENST00000442448.5**).**

**Supplementary Table 1: List of genes covered by NGS panels.**

**1a:** List of 199 genes on Archer Fusion Plex Pan-Heme panel

| *ABL1* | *ABL2* | *AICDA* | *AKT3* | *ALK* | *ASB13* | *ASXL1* | *BATF3* | *BAX* | *BCL11B* |
| --- | --- | --- | --- | --- | --- | --- | --- | --- | --- |
| *BCL2* | *BCL2A1* | *BCL3* | *BCL6* | *BCR* | *BIRC3* | *BLNK* | *BMF* | *BMP7* | *BRAF* |
| *BTK* | *CALR* | *CARD11* | *CBFB* | *CBL* | *CCDC50* | *CCND1* | *CCND2* | *CCND3* | *CD274* |
| *CD44* | *CD79B* | *CDC25A* | *CDK6* | *CDKN2A* | *CDKN2B* | *CEBPA* | *CEBPD* | *CEBPE* | *CEBPG* |
| *CHD1* | *CHIC2* | *CIITA* | *CREB3L2* | *CREBBP* | *CRLF2* | *CSF1R* | *CSF3R* | *CTLA4* | *CYB5R2* |
| *DCK* | *DEK* | *DENND3* | *DLEU1* | *DNM2* | *DNMT3A* | *DNMT3B* | *DNTT* | *DUSP22* | *E2F2* |
| *EBF1* | *EIF4A1* | *ENTPD1* | *EPOR* | *ERG* | *ETV6* | *EXOC2* | *EZH2* | *FAM216A* | *FBXW7* |
| *FGFR1* | *FGFR2* | *FGFR3* | *FLT3* | *FOXP1* | *FUT8* | *GATA1* | *GATA2* | *GLIS2* | *GNAS* |
| *HOXA10* | *HOXA9* | *ID4* | *IDH1* | *IDH2* | *IKZF1* | *IKZF2* | *IKZF3* | *IL16* | *IL7R* |
| *IRF4* | *IRF8* | *ITPKB* | *JAK1* | *JAK2* | *JAK3* | *KAT6A* | *KDM6A* | *KIAA0101* | *KIT* |
| *KLF2* | *KMT2A* | *KRAS* | *LIMD1* | *LMO1* | *LMO2* | *LRMP* | *LYL1* | *LZTS1* | *MAL* |
| *MALT1* | *MAML3* | *MECOM* | *MKL1* | *MLF1* | *MLLT10* | *MLLT4* | *MME* | *MPL* | *MUC1* |
| *MYBL1* | *MYC* | *MYD88* | *MYH11* | *NEK6* | *NF1* | *NFKB1* | *NFKB2* | *NME1* | *NOTCH1* |
| *NOTCH2* | *NPM1* | *NRAS* | *NT5C2* | *NTRK3* | *NUP214* | *NUP98* | *P2RY8* | *PAG1* | *PAICS* |
| *PAX5* | *PBX1* | *PDCD1* | *PDCD1LG2* | *PDGFRA* | *PDGFRB* | *PHF6* | *PICALM* | *PIM1* | *PIM2* |
| *PLCG1* | *PLCG2* | *PML* | *PPAT* | *PRDM16* | *PRKAR2B* | *PTK2B* | *PTPN1* | *PTPN11* | *PYCR1* |
| *RAB29* | *RAG1* | *RAG2* | *RANBP1* | *RARA* | *RBM15* | *RHOA* | *ROS1* | *RUNX1* | *RUNX1T1* |
| *S1PR2* | *SEMA6A* | *SERPINA9* | *SETBP1* | *SETD2* | *SF3B1* | *SH2B3* | *SH3BP5* | *SLC29A1* | *SOX11* |
| *SRSF2* | *STAT3* | *STAT5B* | *STAT6* | *STIL* | *STRBP* | *TAL1* | *TCF3* | *TFG* | *TLX1* |
| *TLX3* | *TNFRSF13B* | *TNFSF4* | *TP63* | *TYK2* | *U2AF1* | *WT1* | *XPO1* | *ZCCHC7* |  |

**Supplementary Table 1b:** List of 157 genes on Archer Pan-Heme VariantPlex NGS panel

| *ABL1* | *ACD* | *AKT3* | *ANKRD26* | *AP3B1* | *ARID1A* | *ASXL1* | *ATM* | *ATRX* | *B2M* |
| --- | --- | --- | --- | --- | --- | --- | --- | --- | --- |
| *BCL10* | *BCL2* | *BCL6* | *BCOR* | *BCORL1* | *BIRC3* | *BRAF* | *BTK* | *CALR* | *CARD11* |
| *CBL* | *CBLB* | *CBLC* | *CCND2* | *CCND3* | *CD274* | *CD79B* | *CDC25C* | *CDKN2A* | *CEBPA* |
| *CECR1* | *CREBBP* | *CSF3R* | *CTC1* | *CUX1* | *CXCR4* | *DARC* | *DCK* | *DDX41* | *DHX15* |
| *DIS3* | *DKC1* | *DNAJC21* | *DNMT3A* | *EFTUD1* | *ELANE* | *EP300* | *ETNK1* | *ETV6* | *EZH2* |
| *FAM46C* | *FBXW7* | *FCGR3B* | *FLT3* | *FOXP1* | *G6PC3* | *GATA1* | *GATA2* | *GFI1* | *GNAS* |
| *HAX1* | *HRAS* | *IDH1* | *IDH2* | *IKZF1* | *IRF4* | *JAGN1* | *JAK2* | *JAK3* | *KDM6A* |
| *KIF23* | *KIT* | *KLF1* | *KMT2A* | *KMT2D* | *KRAS* | *LAT* | *LUC7L2* | *MALT1* | *MAP2K1* |
| *MEF2B* | *MPL* | *MYC* | *MYD88* | *MYSM1* | *NBN* | *NF1* | *NHP2* | *NOP10* | *NOTCH1* |
| *NOTCH2* | *NPM1* | *NRAS* | *PARN* | *PDCD1LG2* | *PDGFRA* | *PHF6* | *PIK3CD* | *PLCG2* | *PPM1D* |
| *PTEN* | *PTPN11* | *RAC2* | *RAD21* | *RB1* | *REL* | *RHOA* | *RUNX1* | *SAMD9* | *SAMD9L* |
| *SETD2* | *SF3B1* | *SH2B3* | *SLC29A1* | *SMARCA4* | *SMC1A* | *SMC3* | *SRP72* | *SRSF2* | *STAG2* |
| *STAT3* | *STAT5B* | *SYK* | *TAZ* | *TCL1A* | *TCN2* | *TERT* | *TET2* | *TINF2* | *TP53* |
| *TP63* | *UBA1* | *U2AF1* | *U2AF2* | *USB1* | *VPS45* | *WAS* | *WHSC1* | *WIPF1* | *WRAP53* |
| *WT1* | *XPO1* | *ZRSR2* | *AMELY* | *ATP13A4* | *DACH2* | *DLEU1* | *DMD* | *ERCC6L2* | *FOXO1* |
| *MAP3K14* | *MEF2BNB-MEF2B* | *PALLD* | *RAB31* | *RMRP* | *RPS14* | *SETBP1* |  |  |  |

The Archer Pan-Heme VariantPlex NGS coverage rate was 98%, the average sequence depth was 2500×, and the sequencing depth of more than 95% of target regions was 2000×. Results were analysed using the Archer Analysis Unlimited Version 7.1.0-14 platform and Ion Torrent Genexus Software 6.6.2.1. Results from the Archer Fusion Plex Pan-Heme panel were analysed using Unlimited platform version 7.4.4.
